# Impact of Drug Administration Routes on the *In Vivo* Efficacy of the Natural Product Sorangicin A Using a *Staphylococcus aureus* Infection Model in Zebrafish Embryos

**DOI:** 10.3390/ijms241612791

**Published:** 2023-08-13

**Authors:** Franziska Fries, Andreas M. Kany, Sari Rasheed, Anna K. H. Hirsch, Rolf Müller, Jennifer Herrmann

**Affiliations:** 1 Helmholtz Institute for Pharmaceutical Research Saarland (HIPS), Helmholtz Centre for Infection Research (HZI), Saarland University, Campus E8 1, 66123 Saarbrücken, Germany; franziska.fries@helmholtz-hips.de (F.F.); andreas.kany@helmholtz-hips.de (A.M.K.); sari.rasheed@helmholtz-hips.de (S.R.); anna.hirsch@helmholtz-hips.de (A.K.H.H.); rolf.mueller@helmholtz-hips.de (R.M.); 2 German Centre for Infection Research (DZIF), Partner Site Hannover-Braunschweig, 38124 Braunschweig, Germany; 3 Department of Pharmacy, Saarland University, 66123 Saarbrücken, Germany

**Keywords:** zebrafish, *Staphylococcus aureus*, infection, drug discovery, *in vivo* efficacy, sorangicin

## Abstract

*Staphylococcus aureus* causes a wide range of infections, and it is one of the leading pathogens responsible for deaths associated with antimicrobial resistance, the rapid spread of which among *S. aureus* urges the discovery of new antibiotics. The evaluation of *in vivo* efficacy of novel drug candidates is usually performed using animal models. Recently, zebrafish (*Danio rerio*) embryos have become increasingly attractive in early drug discovery. Herein, we established a zebrafish embryo model of *S. aureus* infection for evaluation of *in vivo* efficacy of novel potential antimicrobials. A local infection was induced by microinjecting mCherry-expressing *S. aureus* Newman followed by treatment with reference antibiotics via microinjection into different injection sites as well as via waterborne exposure to study the impact of the administration route on efficacy. We successfully used the developed model to evaluate the *in vivo* activity of the natural product sorangicin A, for which common mouse models were not successful due to fast degradation in plasma. In conclusion, we present a novel screening platform for assessing *in vivo* activity at the antibiotic discovery stage. Furthermore, this work provides consideration for the choice of an appropriate administration route based on the physicochemical properties of tested drugs.

## 1. Introduction

The rise of antimicrobial resistance (AMR) poses one of the leading public health threats globally and urges researchers and clinicians to develop novel antibiotics [1,2]. According to recent estimates, AMR could cause as many as 10 million deaths each year by 2050 and, thus, could become the most common cause of death worldwide [3,4]. Despite the AMR spread, many pharmaceutical companies withdraw from antimicrobial research and development (R&D), resulting in a significant decline in the number of novel antibiotics in the pipeline [5,6]. Moreover, most antibiotics that have been approved in recent years and that are currently in development represent analogs of already known classes [7,8,9]. To overcome existing resistance, more new classes of antibiotics are needed, especially those with an unprecedented mode of action. With the aim to raise awareness and support R&D of novel antimicrobial agents, the World Health Organization (WHO) has published a priority pathogens list with *Staphylococcus aureus*, including methicillin-resistant (MRSA), vancomycin-intermediate (VISA) and vancomycin-resistant *S. aureus* (VRSA), as one of the high-priority pathogens [10].

*S. aureus* is an opportunistic pathogen that can cause a wide range of nosocomial and community-acquired infections, such as bacteremia, skin and soft tissue infections, infective endocarditis, and biomaterial-associated infections (BAI) [11,12,13,14]. In 2019, *S. aureus* was one of the six leading pathogens responsible for deaths associated with AMR, in particular, prevalent in high-income countries [1]. The treatment of diseases caused by *S. aureus* is impeded by acquiring immune-evasion strategies and the emergence of (multi)drug-resistant strains. Notably, MRSA is notorious for causing difficult-to-treat infections with high mortality rates in the clinics and also increasingly in the community [15,16,17]. The current treatment regimen for MRSA infections consists of vancomycin and daptomycin as first-line antibiotics. However, in recent years, there has been an increasing rate of vancomycin-resistant (VRSA) and daptomycin-resistant *S. aureus* (DRSA), emphasizing the need for novel antibacterials [18,19,20]. 

During drug discovery, each drug candidate has to be evaluated in terms of *in vivo* efficacy [21]. In recent years, zebrafish (*Danio rerio*) embryos have been used more frequently at various stages of the drug-discovery process as useful and cost-effective alternatives to some mammalian models [22]. Several advantages, such as the optical transparency, the high genetic homology to humans and their small size enabling high-throughput phenotype-based screenings, render the zebrafish a widely used vertebrate model organism [23,24]. 

To date, several *S. aureus* infection models have been established using zebrafish embryos [25]. Even though *S. aureus* is not considered a natural pathogen of the fish, it has been previously reported that it can cause lethal disease in zebrafish embryos and that the bacteria specifically exploit neutrophils to evade killing by the host immune system [26,27]. Using a multi-site infection approach, former studies have shown that the choice of injection site has a significant impact on the level of resistance toward *S. aureus* infection [28].

Herein, we report the development and usage of a local *S. aureus* infection model in zebrafish embryos with the aim of assessing the *in vivo* efficacy of antibiotics. Antibiotic treatment of infected embryos was performed via microinjection into the yolk sac and the caudal vein (CV), as well as via water immersion, to study the impact of the administration route on the efficacy of tested reference drugs. The model was further used to evaluate the *in vivo* efficacy of the myxobacterial natural product sorangicin A [29], a potent macrolide antibiotic for which common mouse models were not successful due to fast degradation in plasma.

## 2. Results

### 2.1. S. aureus Causes a Lethal Infection in Zebrafish Embryos

To visualize bacteria within the zebrafish and quantify bacterial burden, *S. aureus* Newman, a drug-sensitive *S. aureus* strain (Table S1), was transformed with the pRN11 vector enabling the expression of mCherry as fluorescent reporter [30]. The constructed fluorescent reporter strain was characterized *in vitro* in terms of growth and fluorescence intensity. Experiments were carried out at 37 °C, as well as 28 °C, as this temperature is used for maintenance of zebrafish embryos. Compared to the optimal growth temperature at 37 °C, *S. aureus* grows slightly more slowly at 28 °C; however, it reaches comparable maximum cell densities. Fluorescence is delayed by 8 to 9 h as compared to bacterial growth and reaches its maximum in late stationary phase. Importantly, the fluorescence decreases as bacteria enter the decline phase (Figure S1). Thus, any detected fluorescence originates from viable bacteria allowing the usage of fluorescence as a quantitative measure for determination of bacterial burden in the zebrafish model.

**Figure 1:**
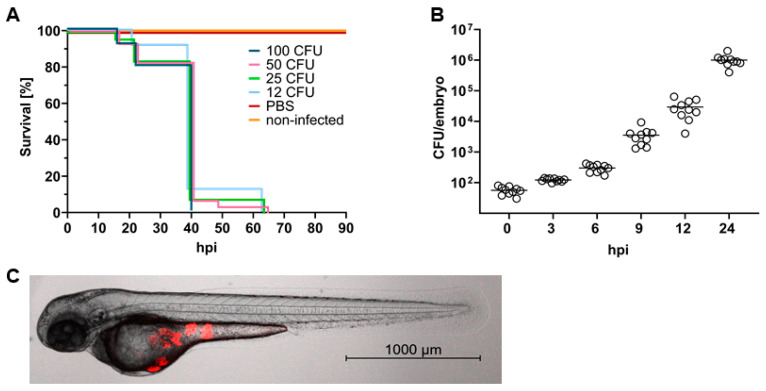
*Staphylococcus aureus* causes a lethal infection in zebrafish embryos. (**A**) Survival of zebrafish embryos infected with rising doses of *S. aureus* Newman via microinjection into the yolk sac. Doses as little as 12 colony-forming units (CFU) cause 100% mortality within 64 h. (**B**) Growth of *S. aureus* within zebrafish embryos upon injection with approximately 50 CFU into the yolk sac. At each time point, 10 living embryos were homogenized, and bacterial numbers were determined (CFU). (**C**) Representative embryo infected with 50 CFU of *S. aureus* Newman. *S. aureus* microinjection leads to local growth of bacteria restricted to the injection site. Image was captured at 24 h post infection (hpi) using the Celldiscoverer 7 with LSM 900 (Zeiss, Jena, Germany) together with the 5×/0.35 Plan-APOCHROMAT objective.

Infection of zebrafish embryos with *S. aureus* was performed at 30 h post fertilization (hpf) via microinjection. In the presented infection model, the yolk was chosen as site of infection since such an approach is easy to perform, allowing high throughput. Furthermore, the yolk was reported to be a site of immune privilege; thus, it has very low resistance to *S. aureus* infection [28], which eases the assessment of *in vivo* efficacy of drugs. To confirm the low resistance of the yolk to staphylococcal infection, embryos were challenged with rising doses of *S. aureus*. As few as 12 colony-forming units (CFU) of *S. aureus* Newman were sufficient to cause a lethal infection, with mortality rates reaching 100% within 64 h, whereas embryos injected with the same volume (4 nL) of sterile phosphate-buffered saline (PBS) survived without any apparent toxic effect. No significant differences between survival rates were observed when assessing infective doses between 12 and 100 CFU (Figure 1A). To assess whether the pathogen is capable of reproducing within the embryos, the *in vivo* growth of *S. aureus* was evaluated. Embryos (30 hpf) were injected into the yolk sac with an intermediate infectious dose of 50 CFU, and at different time points, ten living embryos were homogenized and bacterial numbers were determined (CFU/embryo). *S. aureus* showed logarithmic growth within the zebrafish, reaching bacterial counts greater than 10^6^ CFU/embryo after 24 h (Figure 1B). Injection of *S. aureus* into the yolk leads to local growth of bacteria restricted to the injection site (Figure 1C). At 24 h post infection (hpi), bacteria occurred as fluorescent foci; however, no fluorescence was recorded within the first hours after injection (Video S1).

### 2.2. Experimental Design to Assess In Vivo Efficacy of Drugs against S. aureus in Zebrafish Embryos

With the aim to study the *in vivo* efficacy of drugs, 30 hpf zebrafish embryos were infected with 50 CFU of mCherry-expressing *S. aureus* Newman. Embryos were then left to recover from injection for two hours and subsequently treated with various reference antibiotics. Antibiotic treatment was performed via bath water immersion as well as via microinjection into the yolk (local treatment) and the caudal vein (systemic treatment) to study whether differences in *in vivo* activity exist between the three administration routes. Survival was assessed daily until 120 hpf. Additionally, the infection was visualized and quantified by means of fluorescence microscopy at 24 h post treatment (hpt). At the end of every experiment, living embryos (and dead embryos, if possible) were homogenized to determine the remaining bacterial count. Five approved antibiotics from different classes were assessed *in vivo*. The chosen drugs ought to differ in physicochemical properties, such as molecular weight and polarity, in order to detect a pattern between successful administration routes and the physicochemical properties of the drug. Prior to efficacy assessment, drug-mediated toxicity in zebrafish embryos was investigated using the same administration routes (Figure S3). For microinjection, a fixed dose of 20 to 30 mg/kg was used for each antibiotic. The concentration for bath water immersion was based on the minimum inhibitory concentration (MIC) and the maximum tolerated concentration (MTC) of the drug, whereby it is generally recommended to use 50- to 100-fold the antibacterial MIC to see a positive effect [31,32]. 

### 2.3. Impact of the Route of Administration on Drug Activity

**Figure 2:**
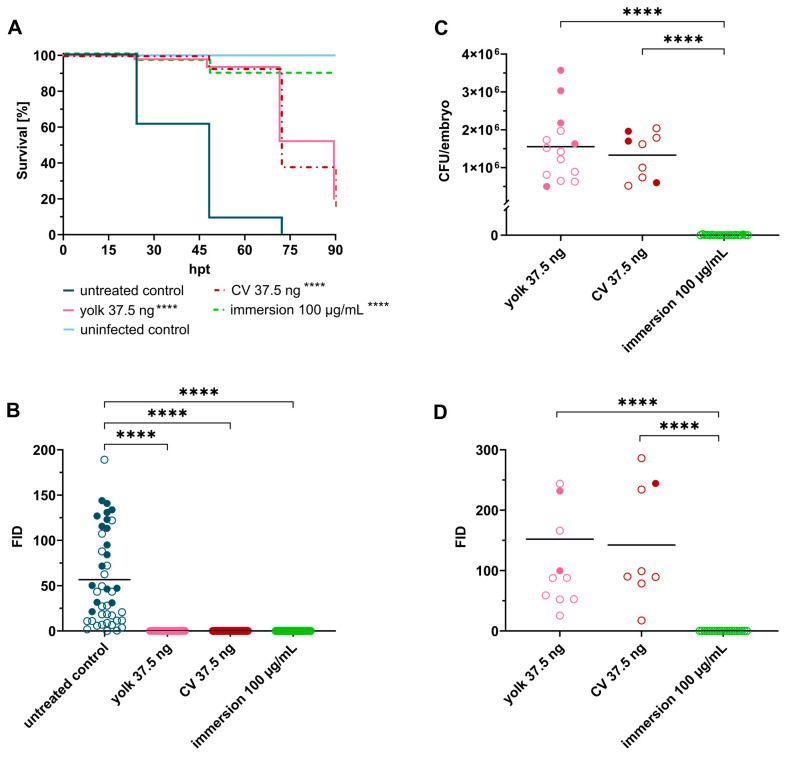
Linezolid treatment significantly prolongs survival of embryos infected with *Staphylococcus aureus*. (**A**) Survival curves of *S. aureus*-infected embryos (≈50 CFU) treated with linezolid at 2 hpi via different administration routes. Non-infected PBS-injected embryos served as negative control. (**B**) Fluorescent Integrated Density (FID) of infected untreated and treated embryos at 24 hpt. (**C**) CFU counts of recovered bacteria from homogenized embryos at 120 hpf. (**D**) FID of linezolid treatment groups at 120 hpf. Open circles represent living embryos, whereas solid circles show dead embryos. CFU: colony-forming unit; CV: caudal vein; *p* < 0.0001: ****.

Linezolid, a synthetic antibiotic belonging to the antimicrobial class of oxazolidinones [33], was the first candidate to be assessed in the *S. aureus* zebrafish embryo model. All treatment protocols significantly (*p* < 0.0001) prolonged the survival of *S. aureus*-infected embryos (Figure 2A). No fluorescence was recorded at 24 hpt in either of the treatment groups (Figure 2B); however, a few individual fish treated via yolk and caudal vein (CV) injection started showing fluorescent foci in their yolk body at 48 hpi, which subsequently matured to become a lethal infection with bacterial numbers exceeding 10^5^ CFU/embryo and mortality rates greater than 75%. In contrast, supplementing the fish water with a high dose of linezolid (50× MIC) led to 90% survival, and more importantly, full recovery from infection was achieved, as determined by fluorescence microscopy and CFU plating (Figure 2C,D). Any dead embryos that occurred in the immersion group were homogenized and plated on agar in order to investigate whether the embryos succumbed to infection or died due to another cause. No bacteria were recovered from these embryos; thus, the embryos were not killed by infection but rather by a potential toxic effect of linezolid (Figure S3). 

**Figure 3:**
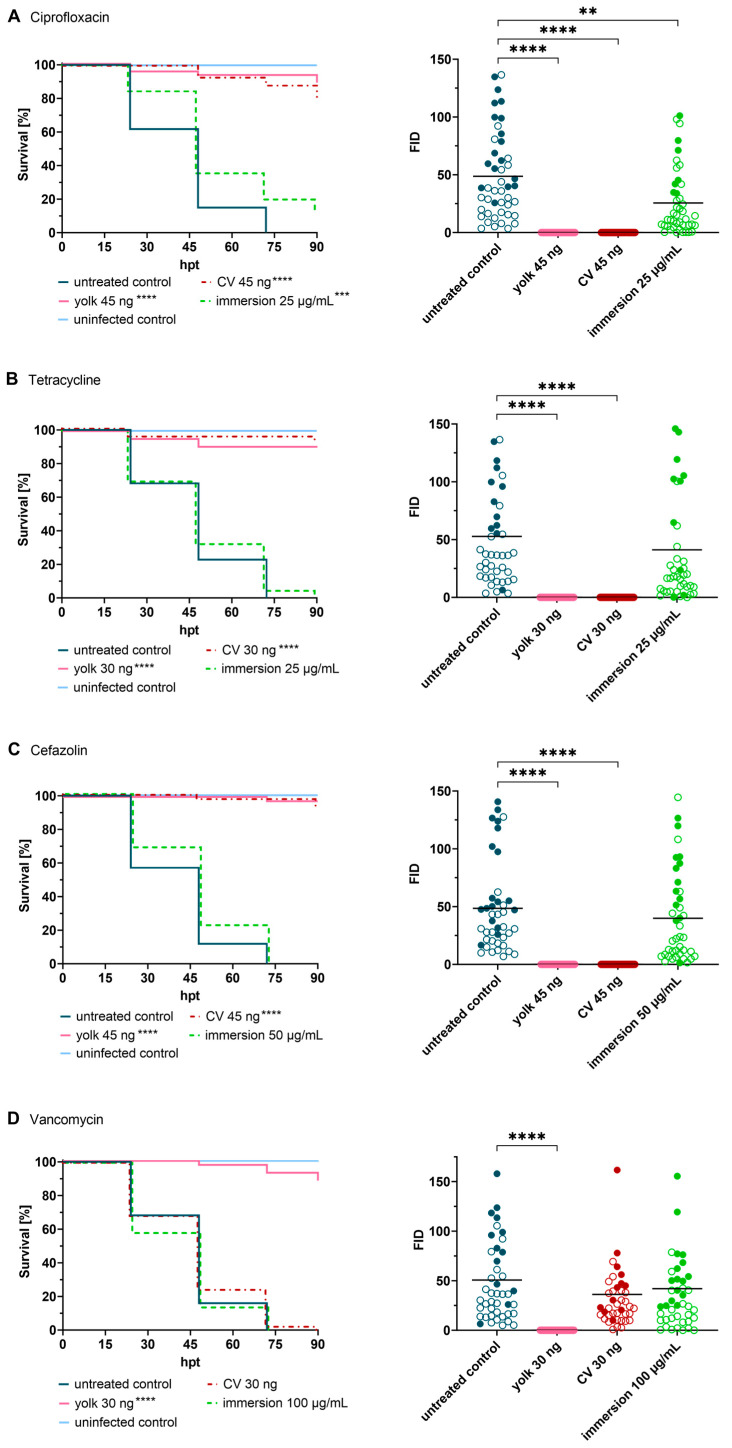
Treatment of infected embryos with reference antibiotics ((**A**) ciprofloxacin, (**B**) tetracycline, (**C**) cefazolin and (**D**) vancomycin). Efficacy of tested drugs was determined by means of survival analysis and quantitative fluorescence microscopy. Survival curves of *Staphylococcus aureus*-infected embryos (≈50 CFU) treated with various reference antibiotics compared to untreated infected embryos (**left panel**), and Fluorescent Integrated Density (FID) of infected and treated embryos (**right panel**) at 24 h post treatment (hpt) are shown. A significant increased survival was detected for each antibiotic in at least one delivery method, however, considerable differences were observed amongst the different administration routes. Open circles represent living embryos, whereas solid circles show dead embryos. CV: caudal vein; *p* < 0.01: **; *p* < 0.001: ***; *p* < 0.0001: ****.

Ciprofloxacin, tetracycline and cefazolin showed a similar activity pattern *in vivo*. All three antibiotics represent rather hydrophilic antibiotics of low molecular weight. They exhibit treatment efficacy when injected directly into the site of infection (yolk) as well as when systemically administered (caudal vein injection, Figure 3). No fluorescence was observed at 24 hpt and unlike linezolid, fluorescence remained absent over the whole course of the experiment. Furthermore, no colonies were recovered at 120 hpf, suggesting total eradication of *S. aureus* inside the embryos. Exposure to these reference antibiotics via bath water failed to protect the embryos from infection. Ciprofloxacin was able to slightly prolong survival and a decrease in FID values was observed at 24 hpt; however, eventually, more than 80% of embryos died due to infection (Figure 3A). Tetracycline and cefazolin did not show any effect when administered via the fish water, as shown in survival rates and FID values equal to the positive control (Figure 3B,C).

Vancomycin represents a high-molecular-weight antibiotic that is considered the gold standard in the treatment of MRSA infections [34]. Therefore, vancomycin was evaluated in the zebrafish model in order to investigate whether such a high molecular weight compound would be able to penetrate embryonic membranes and reach the site of infection. Local treatment with vancomycin prevented embryo death, as reflected in a survival rate almost matching the negative control (90% survival). Furthermore, no development of fluorescence was observed at 24 hpt. At the end of the experiment, few colonies (0–2500 CFU) were recovered from yolk-treated embryos, indicating that small subsets of bacteria were able to escape the bactericidal action of the drug. No increased survival was found when infected embryos were exposed to vancomycin via the fish water and via caudal vein injection. Consistent with the survival curves, there was no reduction in bacterial fluorescence at 24 hpt (Figure 3D). In addition, the efficacy of vancomycin was also not impacted by the time of antibiotic treatment (Figure S2).

### 2.4. In Vivo Evaluation of the Natural Product Sorangicin A

**Figure 4:**
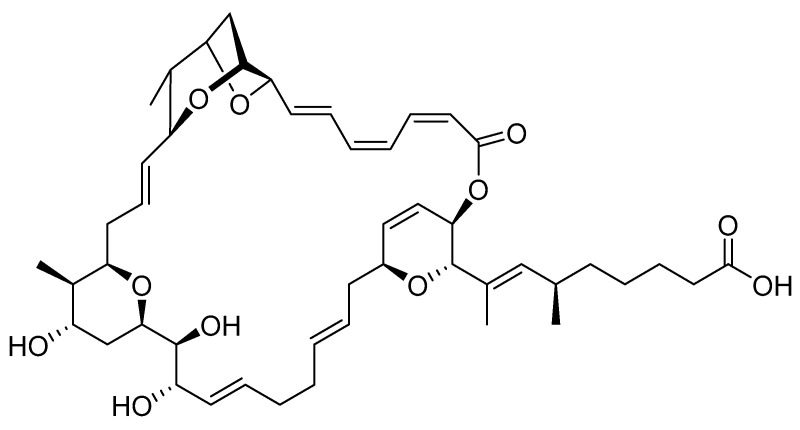
Chemical structure of sorangicin A.

Sorangicin A (SorA) represents a potent macrolide antibiotic derived from the myxobacterium *Sorangium cellulosum* (Figure 4). The natural product shows remarkable activity against Gram-positive bacteria, including mycobacteria such as *M. tuberculosis* (*Mtb*) [29]. SorA inhibits the bacterial RNA polymerase (RNAP) by binding in the same RNAP β-subunit pocket as rifampicin and thereby stalling RNA elongation [35,36]. Previous studies revealed that SorA inhibits a subset of clinically relevant rifampicin-resistant *Mtb* RNAPs by a distinct mechanism prior to chain elongation, as compared to the wild type RNAP. In addition, SorA shows a better pharmacokinetic profile than rifampicin as it displays only moderate CYP3A4 induction, reducing the risk of drug–drug interactions (DDIs) [37]. Given its potential to overcome drug resistance and its strong antibacterial activity against *S. aureus* (Table S1), we aimed to evaluate the natural product *in vivo*.

In former *in vivo* efficacy studies using mouse models of *S. aureus* infection, SorA failed to show a protective effect. Other experimental infection models in rats, on the contrary, were successful (unpublished data from GBF, German Research Centre for Biotechnology; now HZI, Helmholtz Centre for Infection Research), which prompted us to study the pharmacokinetics of SorA in more detail. Metabolic as well as plasma stability were assessed *in vitro* to find key differences between the mouse and rat organisms. SorA shows a short half-life of 3.4 min (Cl_int_ 557 µL/mg/min) upon incubation with mouse liver microsomes, indicating rapid metabolic degradation. Metabolic stability of SorA in rats was similarly poor (t_1/2_ 4.4 min, CL_int_ 239 µL/mg/min), suggesting that metabolic degradation is most likely not the main cause for the disparity between mouse and rat infection models. When investigating the plasma stability, we found that SorA is quickly degraded in mouse plasma. The compound has a half-life of 17.5 min, and after 4 h, the plasma concentration of SorA was below the limit of detection. In contrast, SorA was much more stable in rat and human plasma. In plasma protein binding assays, SorA showed little interspecies variation with a sufficiently high free fraction (> 10%) in all tested species (Table 1). Taking these findings into consideration, we concluded that the rat represents the more suitable rodent species for *in vivo* pharmacodynamic (PD) studies of SorA. 

To confirm these findings *in vivo*, a pharmacokinetic (PK) study was performed in male CD-1 mice. For this, nine mice were administered 5 mg/kg of SorA intravenously, and plasma concentrations were determined over 7 h. Following *i.v.* administration, the compound showed a short half-life of 0.87 h combined with a total clearance of 14,287.3 mL/h/kg and a low systemic exposure (AUC 340.9 ng/mL·h, Table 2). The plasma levels of SorA were maintained above the MIC of *S. aureus* for less than 30 min (Figure S5), providing a possible explanation for the failure in mouse PD models.

Having confirmed *in vitro* and *in vivo* that the mouse is not a good model to study the antibacterial efficacy of SorA, we set out to demonstrate the utility and even perhaps the superiority of the zebrafish embryo model. The latter is not considered as an animal experiment and, thus, poses much less of an ethical concern than testing in higher species such as rat and rabbit. In addition, the zebrafish embryo model requires only minute amounts of compound, whereas the compound demand for larger rodent models can easily reach the gram scale. Prior to assessing *in vivo* activity, we studied the safety of SorA in zebrafish embryos. The natural product was well-tolerated in concentrations up to 100 µg/mL upon short- as well as long-term aquatic exposure. Solely long-term exposure to the highest concentration (200 µg/mL, equals 3.200× the MIC) led to a 10% reduction in embryo survival with respect to the control group (1% DMSO). Likewise, the injection dose of 30 mg/kg was well-tolerated by the zebrafish embryos and did not induce any pathophysiological symptoms or malformations (Figure S4). 

**Figure 5:**
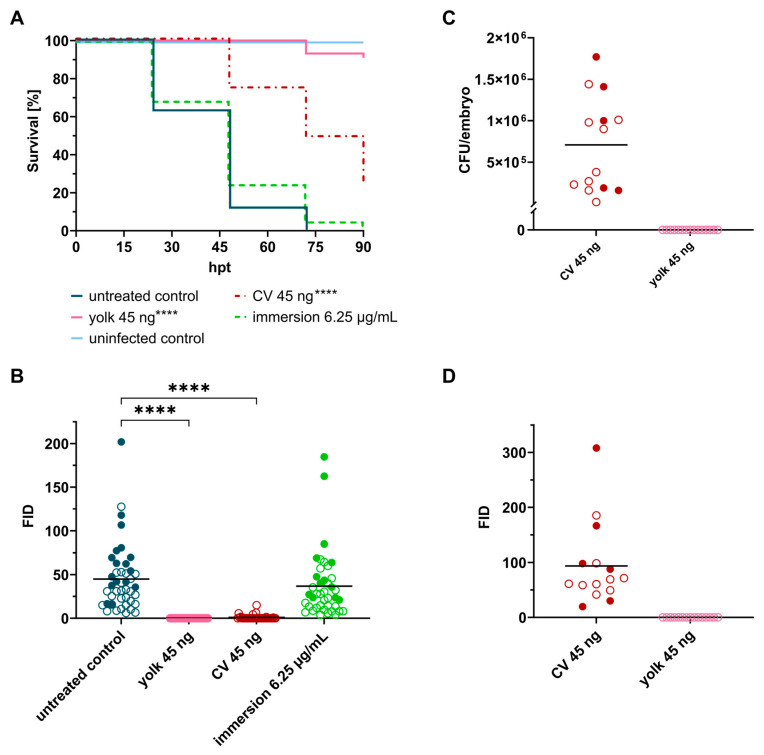
Sorangicin A administered via yolk microinjection significantly prolongs embryo survival and leads to full recovery of zebrafish embryos. (**A**) Survival curves of *Staphylococcus aureus*-infected embryos treated with sorangicin A at 2 hpi via different administration routes. Non-infected PBS-injected embryos served as negative control. (**B**) Fluorescent-integrated density (FID) of infected untreated and treated embryos at 24 hpt. (**C**) CFU counts of recovered bacteria from homogenized embryos at 120 hpf. (**D**) FID of microinjection treatment groups at 120 hpf. Open circles represent living embryos, whereas solid circles show dead embryos. CV: caudal vein; *p* < 0.0001: ****.

Having shown that SorA is safe in the zebrafish embryo model, we proceeded with the study of *in vivo* efficacy. Microinjection of SorA into the yolk sac of *S. aureus*-infected embryos significantly increased the survival rate (*p* < 0.0001, Figure 5A) and reduced bacterial burden as determined by fluorescence microscopy (Figure 5B). CFU determination at 120 hpf revealed that the antibiotic treatment led to complete eradication of bacteria and, thus, curation of infection (Figure 5C). Systemic treatment via microinjection into the CV also significantly extended the lifespan of infected fish (*p* < 0.0001, Figure 5A). Bacterial fluorescence was suppressed at 24 hpt (Figure 5B); however, fluorescence increased over the following three days (Figure 5D), and a high percentage of embryos (75%) eventually succumbed to the infection with bacterial numbers exceeding 10^4^ CFU/embryo (Figure 5C). Upon bath water immersion, SorA did not show any protective effect on infected embryos. Even after supplementing the bath water with 1000× MIC, no difference was noticed between treated and untreated embryos.

## 3. Discussion

Zebrafish embryos represent an attractive *in vivo* model to study infectious diseases as already applied for various microorganisms, including organisms that are usually not considered natural pathogens of the fish, such as *S. typhimurium* [38,39,40] or the here-described *S. aureus* [26,28]. Since the introduction of the 3R principle [41], researchers have strived to find alternatives to classical animal models. In this context, a couple of non-mammalian hosts have emerged in recent years; mainly invertebrates such as *Caenorhabditis elegans*, *Galleria mellonella*, *Drosophila melanogaster* and *Bombyx mori*. These invertebrate models provide several advantages, for instance, easy maintenance, low costs and the usage as a high-throughput screening model [42]. However, they are only useful to a limited extent as their physiology and immune systems differ significantly from mammalian models [43]. The zebrafish, in contrast, possesses functional organs, shares high genetic homology with humans [24] and developed both innate and acquired immune systems [44]. Furthermore, many advantages of invertebrates also apply to the zebrafish, rendering the vertebrate an excellent alternative for studying infectious disease biology as well as for high-throughput *in vivo* drug screening.

Prajsnar and colleagues have already confirmed that zebrafish embryos represent a useful tool for studying the pathogenesis of staphylococcal infection. In their natural habitat, zebrafish are unlikely to have prior exposure to *S. aureus*, yet it could be shown that the pathogen is capable of causing a lethal infection upon systemic administration as well as local yolk sac injection. While in the systemic setting, the zebrafish showed phagocyte-dependent resistance to *S. aureus*, the yolk was highly susceptible to staphylococcal infection. When injecting *S. aureus* into the yolk of wild type and myeloid-depleted embryos, Prajsnar *et al.* found that survival rates were equivalent, suggesting that the yolk may be a site of immune privilege. This may be due to the physical nature of the yolk, hindering both the sensing of bacteria as well as chemotaxis of phagocytes into the site of infection [26].

To date, a handful of studies used *S. aureus*-infected zebrafish embryos to assess *in vivo* efficacy of new antimicrobials [25]. However, in contrast to already published studies, the herein presented model provides full validation with a series of reference antibiotics. Due to the little information available concerning the uptake of drugs via passive diffusion through the skin and the failure of some compounds to reduce the bacterial burden in zebrafish embryos when added to the fish water [45], different administration routes were used to deliver the drugs. Besides water exposure, antibiotics were directly injected into the fish at the site of infection (yolk) and into the systemic circulation (caudal vein). 

In accordance with published *S. aureus* zebrafish infection models [26,28], small inoculums of *S. aureus* were sufficient to cause 100% mortality upon yolk sac infection. Here, an intermediate infectious dose of 50 CFU of *S. aureus* Newman led to 100% mortality within 70 h, providing a suitable time window for the assessment of antibiotic activity. Linezolid was shown to exert its maximum therapeutic effect when administered via waterborne exposure. The drug is of low molecular weight (MW: 337.35 g/mol) and of lipophilic nature, hence, it shows favorable physicochemical properties regarding passive diffusion through the skin. Ordas *et al.* previously reported a strong positive correlation between drug hydrophobicity and uptake levels, the latter of which often represents an obstacle in zebrafish embryo models, leading to the failure of compounds to decrease bacterial load [46]. Furthermore, linezolid was capable of reducing bacterial burden temporarily when injected into the caudal vein and the yolk sac. A possible explanation for this limited efficacy is that linezolid only exerts bacteriostatic action, *i.e*., the antibiotic inhibits bacterial growth and requires phagocytic cells in order to definitely eradicate the bacteria. Given the immune privilege of the yolk [26], it may be that linezolid only suppressed bacterial growth initially but was not able to kill the bacteria in the absence of professional phagocytes, allowing subsequent bacterial dissemination and death of zebrafish embryos. In the future, it might be interesting to study whether other bacteriostatic drugs show the same behavior in yolk-infected zebrafish embryos.

Ciprofloxacin, tetracycline and cefazolin represent hydrophilic antibiotics of low molecular weight. Each of the compounds was active *in vivo* when injected into the zebrafish embryos. Unlike linezolid, these three drugs are bactericidal (tetracycline exhibits bactericidal activity against *S. aureus* at concentrations above the MIC [47]); thus, they are independent of the immune system, enabling clearance of the local infection. When supplementing the fish water with high concentrations of the antibiotics, no differences were observed compared to infected untreated embryos. It appears likely that impaired drug uptake may be the cause for this failure. Given the small size of the drugs, molecular weight can be excluded as the principal reason for the limited uptake. The latter is rather affected by the polarity of the drugs as they represent ionic compounds. According to toxicokinetic studies performed by Brox *et al*., uptake rates of charged compounds are much lower compared to those of nonionic compounds, reflecting that diffusion of ionic compounds is hindered [48]. To overcome this issue, infection and treatment studies may be performed at later developmental stages, as embryos show increasing drug sensitivity with age [49,50]. For example, shifting the approach from 30 hpf to 72 hpf could make use of the fact that embryos start to swallow [51]; thus, the uptake of drugs is not only dependent on passive diffusion through the skin but can also be realized by absorption from the gastrointestinal tract. Generally, drugs can also be taken up through the gills of larvae. However, gill respiration in larvae is only present after 12 days post fertilization (dpf) [52]; consequently, the zebrafish model would then become a proper animal model (larvae older than 120 hpf).

Failure of the bath water immersion route was also observed for vancomycin. Besides being a charged molecule, vancomycin (MW: 1449.2 g/mol) is significantly larger than the other tested antibiotics. It is, therefore, not surprising that the outcome of aquatic exposure was negative. In addition, the antibiotic also failed to reach the site of infection when administered into the systemic circulation. This is most likely a consequence of the high molecular weight as well, hindering the drug from crossing biological membranes. This circumstance is also known in humans as orally-administered vancomycin is scarcely absorbed from the gastrointestinal tract and, therefore, increasingly being utilized in the treatment of severe *Clostridioides difficile* colitis [53]. 

Taken together, we can confirm that the route of drug administration has a great impact on *in vivo* efficacy of antibiotics and that the physicochemical properties of tested drugs provide valuable information on whether a certain drug exposure route is useful. Each of the reference antibiotics showed *in vivo* activity in at least one of the used administration routes, demonstrating a proof of concept of the presented model for drug efficacy testing. 

This being the case, we aimed to assess the *in vivo* activity of the antibacterial natural product sorangicin A, for which standard mouse models of infection failed to show *in vivo* efficacy due to insufficient ADME/PK properties in this species. Considering the high homology to humans [24] and the high concordance with respect to drug metabolism [54], the *in vitro* PK data give rise to the assumption that the zebrafish is the better-suited PD model organism in the case of the RNAP inhibitor sorangicin A. SorA showed complete curation of the infection when zebrafish embryos were locally treated, reflecting its strong bactericidal activity against *S. aureus*. Systemic treatment also had a positive impact on embryo survival; however, it only extended the lifespan of infected fish and was not able to eradicate the bacteria. This may be due to several reasons: (1) *in vitro* studies revealed rapid metabolic degradation in different species, which could also lead to reduced concentrations at the site of infection in zebrafish; however, considering that the embryonic liver is only functional by 4 dpf [55], metabolic degradation is of rather secondary importance; (2) it cannot be ruled out that SorA shows instability in zebrafish plasma as it was shown *in vitro* for the mouse and (3) plasma-protein binding may lead to insufficient biodistribution. While the latter can be assessed by mass-spectrometry imaging [54], it is challenging to measure plasma concentrations in zebrafish embryos. Initial studies using nanoscale blood sampling have been performed in 5 dpf embryos [56]; however, equivalent approaches in younger embryos (1–2 dpf) are still lacking. Aquatic exposure of infected embryos to SorA had, as already expected, considering the physicochemical properties of the drug, no positive effect on bacterial load or embryo survival. Although SorA represents a lipophilic molecule, suggesting sufficient uptake levels, at physiological conditions, the carboxyl group of the compound is mostly deprotonated. The ionic character of the compound, as well as the molecular weight being relatively large (MW: 807.03 g/mol), represent unfavorable properties with respect to diffusion through the skin, thus, leading to failure of the aquatic exposure route.

In conclusion, we present a zebrafish embryo model of *S. aureus* infection that can be used to assess drug *in vivo* activity at an early stage during preclinical development, as shown in the example of SorA, where zebrafish embryos were superior to mouse models. It is noteworthy that the zebrafish might never fully replace studies in higher animals prior to first-in-human trials; however, it can be used to complement conventional *in vivo* infection models such as mice or rats. The treatment failure of the first-line drug vancomycin after systemic administration demonstrates some limitations of the applied infection model. Despite being highly effective in human therapy (intravenous administration) to treat infections caused by *S. aureus*, the drug obviously could not reach the site of infection in the zebrafish embryos. In turn, a negative outcome in terms of drug efficacy after caudal vein injection or waterborne exposure does not necessarily translate into a lack of efficacy in other animal models or humans. Similarly, administration of drugs into the yolk sac might also lead to false negatives for e.g., highly lipid-bound drugs, highlighting the importance of a suitable drug administration route to diminish such risks.

## 4. Materials and Methods

### 4.1. Zebrafish Lines and Maintenance

Husbandry of adult zebrafish was performed according to internal guidelines set out in the German Animal Welfare Act (§11 Abs. 1 TierSchG). Experiments were carried out with wild type AB (obtained from the European Zebrafish Resource Center at Karlsruhe Institute of Technology) embryos within the first 120 h post-fertilization (hpf) as these early life stages are not considered as animal experiments according to the EU Directive 2010/63/EU [57]. Embryos were maintained in fresh 0.3× Danieau’s (17.4 mM NaCl, 0.21 mM KCl, 0.12 mM MgSO_4_, 0.18 mM Ca(NO_3_)_2_, 1.5 mM HEPES, 1.2 µM methylene blue, pH 7.1–7.3) at 28 °C. At a maximum of 120 hpf, embryos were euthanized by submersion in ice water for at least twelve hours.

### 4.2. Minimum Inhibitory Concentration (MIC)

Cefazolin was obtained from Acros Organics. Ciprofloxacin hydrochloride, linezolid and vancomycin hydrochloride were obtained from Sigma-Aldrich (St. Louis, MO, USA). Tetracycline hydrochloride was obtained from Alfa Aesar. Sorangicin A was provided by HZI, Braunschweig. Stock solutions were prepared in Milli-Q water or dimethyl sulfoxide (DMSO, Sigma-Aldrich). Minimum inhibitory concentrations (MICs) were determined in cation-adjusted Mueller-Hinton broth (MHB2) using the broth microdilution method as recommended by the Clinical and Laboratory Standards Institute (CLSI). The MIC was defined as the lowest concentration of the antibiotic causing complete inhibition of visible growth of the microorganism (CLSI, 2017) [58]. 

### 4.3. Staphylococcus aureus Transformation 

Plasmid pRN11 (Addgene, Watertown, MA, USA) was extracted from growth strain *E. coli* DC10b using the GeneJET Plasmid Miniprep Kit (Thermo Scientific, Waltham, MA, USA). Preparation of electrocompetent cells and transformation of *S. aureus* Newman was performed as previously described [59]. 

### 4.4. In Vitro Growth Analysis of S. aureus 

*S. aureus* Newman mCherry was grown overnight in Tryptic Soy Broth (TSB, Sigma-Aldrich) supplemented with 10 µg/mL chloramphenicol (Sigma-Aldrich) at 37 °C. The next morning, the culture was diluted to an OD_600_ of 0.3 and reincubated until mid-logarithmic phase (OD_600_ 0.6–0.8) was reached. Bacterial proliferation (λ = 600 nm) and fluorescence (λ_excitation_ = 580 nm, λ_emission_ = 610 nm) were analyzed at 28 °C and 37 °C using a plate reader (Infinite M200 Pro, Tecan Group Ltd., Männedorf, Switzerland). 

### 4.5. Preparation of Bacterial Microinjection Stock Solutions

*S. aureus* Newman mCherry was grown overnight in Tryptic Soy Broth (TSB) supplemented with 10 µg/mL chloramphenicol at 37 °C. The overnight culture was diluted to an OD_600_ of 0.3 and reincubated until mid-logarithmic phase (OD_600_ 0.6–0.8) was reached. The bacterial suspension was centrifuged (RT, 4000 rpm, 10 min), washed twice with sterile phosphate-buffered saline (PBS), resuspended in 4% (*m*/*v*) polyvinylpyrrolidone 40 (PVP 40) in PBS and diluted to the desired cell count. 50 µL aliquots were stored at −80 °C.

### 4.6. Microinjection of Zebrafish Embryos

Pulled glass capillaries for microinjection (Table S2) were prepared using a micropipette puller (P-1000, Sutter Instrument). Dechorionated and anaesthetized embryos were infected with 50 colony-forming units (CFU) of mCherry-expressing *S. aureus* Newman by microinjection into the yolk sac at 30 hpf. In order to confirm bacterial numbers, an equal volume of bacterial cells was ejected onto Tryptic Soy Agar (TSA) supplemented with 10 µg/mL chloramphenicol before and after the injection. Antibiotic treatment was performed two hours post infection (hpi) via three different administration routes, namely bath water immersion and microinjection into the yolk sac as well as the caudal vein (CV). The infection procedure and treatment of infected embryos are described in details in the supplementary materials (Table S3).

### 4.7. Toxicity of Antimicrobials on Zebrafish Embryos

Toxicity of antibiotics was assessed by aquatic exposure and microinjection. For evaluation of the maximum tolerated concentration (MTC) by aquatic exposure, embryos were dechorionated at 30 hpf and placed in a flat-bottom 96-well plate (Sarstedt) with one embryo per well. Excess medium was removed and 150 µL of compound dilutions (in 0.3× Danieau’s, maximum of 1% DMSO) were added. For each antibiotic, a range of concentrations was investigated. Ten embryos were used per concentration and ten additional embryos incubated in fish medium without any antibiotic served as negative control. Microinjection of antibiotics into the caudal vein (CV) and the yolk sac was performed as described earlier. PBS-injected embryos served as negative control. Exposed embryos were maintained at 28 °C throughout the whole experiment. Embryos were monitored daily under a stereo microscope (Stemi 508, Zeiss) in order to record survival as well as anomalies, pigmentation, heartbeat and locomotor responses. An embryo was considered dead when no heart beat could be observed.

### 4.8. Determination of Bacterial Burden

Embryos were transferred individually to microcentrifuge tubes and washed at least three times with sterile PBS. 200 µL of sterile PBS were added and embryos were mechanically homogenized using a micropestle (Carl Roth, Karlsruhe, Germany). 800 µL of sterile PBS were added to reach a final volume of 1 mL. The homogenates were diluted and plated on tryptic soy agar (TSA). CFU counts were determined after 24 h of incubation at 37 °C.

### 4.9. Imaging of Infected Zebrafish Embryos

Embryos were placed in a black 96-well plate (Falcon; Thermo Fisher, Dreieich, Germany) and cryo-anaesthetized by submersion into ice water. Images for quantitative fluorescence analysis were acquired using the following equipment: a fluorescence stereo microscope (M205 FA, Leica Microsystems, Wetzlar, Germany) in combination with a fluorescence illuminator (X-Cite, 200DC, Excelitas Technologies, Waltham, MA, USA) and a microscope camera (DFC7000 T, Leica Microsystems). Quantification of fluorescence was performed using Fiji ImageJ version 2.1.0. Fluorescent Integrated Density (FID) was determined by counting the number of pixels with fluorescence intensity greater than the threshold (10) and multiplying the count with its mean intensity. For confocal imaging, embryos were anaesthetized through immersion in tricaine (945 µM, Sigma-Aldrich) and immobilized in 1.2% low-melting agarose in a flat-bottom 96-well plate. Confocal images and time-lapse series were acquired using the Celldiscoverer 7 with LSM 900 (Zeiss) together with the 5×/0.35 Plan-APOCHROMAT objective.

### 4.10. Statistical Analysis

Statistical analysis was performed using GraphPad Prism version 9.1.0. Survival experiments were evaluated using the Kaplan-Meier method. Comparison between survival curves were made using the log rank (Mantel-Cox) test. For comparison of Fluorescent Integrated Density (FID) values and CFU numbers between two groups, an ordinary one-way ANOVA with multiple comparisons was performed. Statistical significance was assumed at *p*-values below 0.05 (*p* < 0.05: *, *p* < 0.01: **, *p* < 0.001: ***, *p* < 0.0001: ****).

### 4.11. In Vitro Pharmacokinetic Profiling

For the evaluation of phase I metabolic stability, the compound (1 µM) was incubated with 0.5 mg/mL pooled mouse/rat liver microsomes (Xenotech, Kansas City, MO, USA) or human liver microsomes (Corning, NY, USA), 2 mM NADPH and 10 mM MgCl_2_ at pH 7.4 for 120 min at 37 °C. The metabolic stability of testosterone, verapamil and ketoconazole were determined in parallel to confirm the enzymatic activity of mouse/rat liver microsomes; for human liver microsomes testosterone, diclofenac and propranolol were used. The incubation was stopped after defined time points by precipitation of aliquots of the assay mixture with two volumes of ice-cold acetonitrile containing internal standard (15 nM diphenhydramine). Samples were stored on ice until the end of the incubation and precipitated protein was removed by centrifugation (15 min, 4 °C, 4000× *g*). The concentration of the remaining sorangicin A at the different time points was analyzed by HPLC-MS/MS (TSQ Altis Plus, Thermo Fisher, Dreieich, Germany) and used to determine half-life (t_1/2_) and intrinsic clearance (CL_int_).

To determine stability in plasma, a similar setup as for the determination of metabolic stability was applied using pooled mouse, human or rat plasma (Neo Biotech, Nanterre, France). Samples were taken by mixing aliquots with four volumes of ice cold acetonitrile containing internal standard (12.5 nM diphenhydramine). The plasma stability of procain, propantheline and diltiazem were determined in parallel to confirm the enzymatic activity.

Plasma protein binding was determined using the Rapid Equilibrium Dialysis (RED) system (Thermo Fisher Scientific, Waltham, MA, USA). The compound was diluted in pooled murine (CD-1)/human or Wistar rat plasma (Neo Biotech, Nanterre, France) to 10 µM and added to the respective chamber according to the manufacturer’s protocol, followed by addition of PBS pH 7.4 to the opposite chamber. Samples were taken immediately after addition to the plate as well as after 2, 4 and 5 h by mixing 10 µL with 80 µL ice-cold acetonitrile containing 12.5 nM diphenhydramine as internal standard, followed by addition of 10 µL plasma to samples taken from PBS and vice versa. Samples were stored on ice until the end of the incubation and precipitated protein was removed by centrifugation (15 min, 4 °C, 4000× *g*, 2 centrifugation steps). Concentration of the remaining test compound at the different time points was analyzed by HPLC-MS/MS (TSQ Altis Plus, Thermo Fisher, Dreieich, Germany). The amount of compound bound to protein was calculated using the equation PPB [%] = 100 − 100 × (amount in buffer chamber/amount in plasma chamber).

### 4.12. In Vivo Pharmacokinetics in Mice

Pharmacokinetic evaluation of sorangicin A in mice was carried out by Pharmacelsus GmbH (Saarbrücken, Germany). All mouse experiments were approved by and conducted in accordance with the regulations of the local Animal Welfare authorities. Nine adult male CD-1 mice (purchased from Janvier Labs, Le Genest-Saint-Isle, France) were used. Animals were housed in a separate temperature-controlled room (20–24 °C) and maintained in a 12 h light/12 h dark cycle. Sorangicin A was dissolved in DMSO/Cremophor EL/0.9% saline (10:10:80 *v*/*v*) and administered intravenously (dose 5 mg/kg, application volume 5 mL/kg). Retrobulbar blood samples were collected from three animals at 0.083, 0.25, 0.5, 1, 3 and 7 h after *i.v.* administration (two time points/ animal). At each of the designated time points 100 µL blood was collected from the eye into lithium heparin tubes. Quantification of sorangicin A in the generated plasma was performed by LC-MS/MS analysis. The pharmacokinetic analysis was performed by applying a non-compartment model using the Kinetica 5.0 software (Thermo Scientific, Waltham, MA, USA). All given parameters were obtained by trapezoid area calculation.

## Figures and Tables

**Table 1:** *In vitro* pharmacokinetic profiling of sorangicin A. Sorangicin A shows species-dependent metabolic instability and degradation in mouse plasma. t_1/2_: half-life; CL_int_: intrinsic clearance; PPB: plasma protein binding.

Species	Metabolic Stability		Plasma Stability	PPB (%)
	Liver Microsomes t_1/2_ (min)	CL_int_ (µL/mg/min)	t_1/2_ (min)	
mouse	3.4 ± 2.6	557 ± 297	17.5 ± 7.1	87.9 ± 4.8
rat	4.4 ± 0.5	239 ± 155	> 240	88.8 ± 1.7
human	15.7 ± 2.8	90.4 ± 16.5	> 240	87.0 ± 0.4

**Table 2:** *In vivo* pharmacokinetics (PK) of sorangicin A in male CD-1 mice after intravenous administration of 5 mg/kg. c_0_: maximum blood concentration based on the extrapolated time point zero value; c_z_: last measured blood concentration; t_1/2z_: half-life during terminal phase; AUC(0-t_z_): area under the concentration-time curve from time point zero to the last measured time point; V_z_: volume of distribution during terminal phase; CL: total clearance.

PK Parameters: 5 mg/kg *i.v.*	
c_0_ (ng/mL)	1833.6
c_Z_ (ng/mL)	7.2
t_1/2_ (h)	0.87
AUC(0-t_z_) (ng·h/mL)	340.9
V_z_ (mL/kg)	17,961.8
CL (mL/h/kg)	14,287.3

## Data Availability

The data presented in this study are available in [article and supplementary material].

## Supplementary Materials

The following supporting information can be downloaded at: https://www.mdpi.com/1422-0067/24/16/12791/s1.
