# Correction: Fries et al. Impact of Drug Administration Routes on the *In Vivo* Efficacy of the Natural Product Sorangicin A Using a *Staphylococcus aureus* Infection Model in Zebrafish Embryos. *Int. J. Mol. Sci.* 2023, *24*, 12791

**DOI:** 10.3390/ijms25042011

**Published:** 2024-02-07

**Authors:** Franziska Fries, Andreas M. Kany, Sari Rasheed, Anna K. H. Hirsch, Rolf Müller, Jennifer Herrmann

**Affiliations:** 1 Helmholtz Institute for Pharmaceutical Research Saarland (HIPS), Helmholtz Centre for Infection Research (HZI), Saarland University, Campus E8 1, 66123 Saarbrücken, Germany; franziska.fries@helmholtz-hips.de (F.F.); andreas.kany@helmholtz-hips.de (A.M.K.); sari.rasheed@helmholtz-hips.de (S.R.); anna.hirsch@helmholtz-hips.de (A.K.H.H.); rolf.mueller@helmholtz-hips.de (R.M.); 2 German Centre for Infection Research (DZIF), Partner Site Hannover-Braunschweig, 38124 Braunschweig, Germany; 3 Department of Pharmacy, Saarland University, 66123 Saarbrücken, Germany

The authors would like to make the following corrections to the original publication [1]. An incorrect version was released due to a production error. During the revision process, one reviewer suggested to convert Table 1 of the original submission [1] into a figure and to change the colors in the graphs. The authors followed these suggestions and re-modeled Table 1 into Figures 2 and 3. As a consequence of the introduced changes, the numbering of the figures and tables changed in the text. Because of the re-modeling of Table 1 to Figures 2 and 3, the reference list was updated. The corrected Figure 2 and Figure 3 appear below. The authors state that the scientific conclusions are unaffected. This correction was approved by the Academic Editor. The original publication has also been updated.

## Figures and Tables

**Figure 2:**
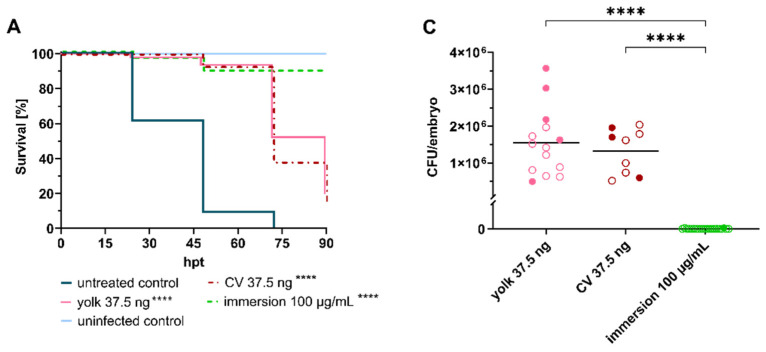

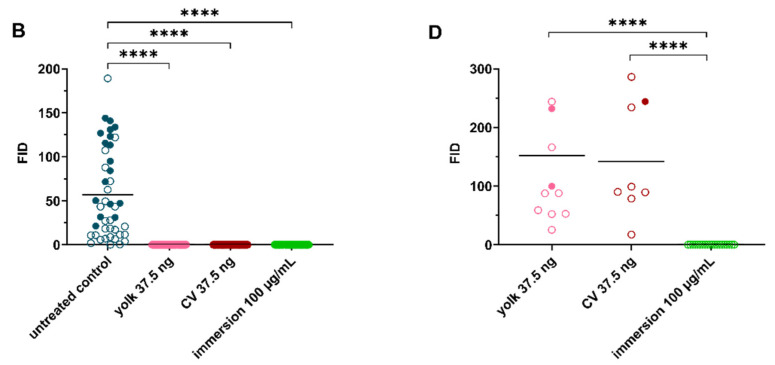
Linezolid treatment significantly prolongs survival of embryos infected with *Staphylococcus aureus*. (**A**) Survival curves of *S. aureus*-infected embryos (≈50 CFU) treated with linezolid at 2 hpi via different administration routes. Non-infected PBS-injected embryos served as negative control. (**B**) Fluorescent Integrated Density (FID) of infected untreated and treated embryos at 24 hpt. (**C**) CFU counts of recovered bacteria from homogenized embryos at 120 hpf. (**D**) FID of linezolid treatment groups at 120 hpf. Open circles represent living embryos, whereas solid circles show dead embryos. CFU: colony-forming unit; CV: caudal vein; *p* < 0.0001: ****.

**Figure 3:**
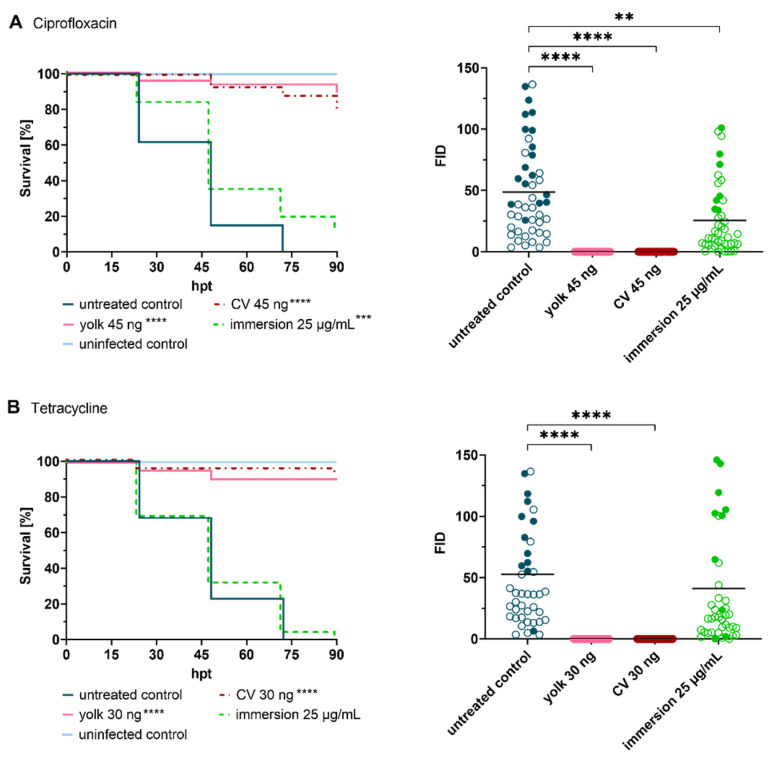

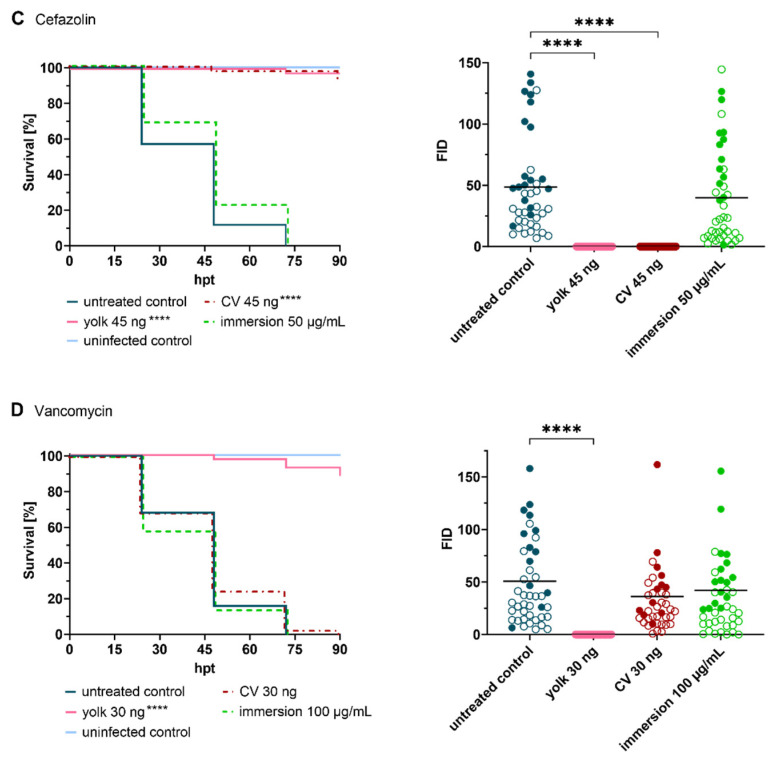
Treatment of infected embryos with reference antibiotics ((**A**) ciprofloxacin, (**B**) tetracycline, (**C**) cefazolin and (**D**) vancomycin). Efficacy of tested drugs was determined by means of survival analysis and quantitative fluorescence microscopy. Survival curves of *Staphylococcus aureus*-infected embryos (≈50 CFU) treated with various reference antibiotics compared to untreated infected embryos (**left panel**), and Fluorescent Integrated Density (FID) of infected and treated embryos (**right panel**) at 24 h post treatment (hpt) are shown. A significant increased survival was detected for each antibiotic in at least one delivery method, however, considerable differences were observed amongst the different administration routes. Open circles represent living embryos, whereas solid circles show dead embryos. CV: caudal vein; *p* < 0.01: **; *p* < 0.001: ***; *p* < 0.0001: ****.
