# Supplementary material for: Impact of Drug Administration Routes on the In Vivo Efficacy of the Natural Product Sorangicin A Using a Staphylococcus aureus Infection Model in Zebrafish Embryos
Source: Int J Mol Sci. 2023 Aug 13;24(16):12791. doi: 10.3390/ijms241612791 (PMC10454396; doi:10.3390/ijms241612791)
Supplement: Supplementary file 1 [file ijms-24-12791-s001.zip › ijms-2501900-supplementary.pdf]

Supporting Information

Impact of Drug Administration Routes on the *in vivo* Efficacy of the Natural Product Sorangicin A using a *Staphylococcus aureus* Infection Model in Zebrafish Embryos

*Franziska Fries, Andreas M. Kany, Sari Rasheed, Anna K. H. Hirsch, Rolf Müller and Jennifer Herrmann**

1. *In vitro* characterization of mCherry expressing *Staphylococcus aureus* Newman

1.1 Minimum inhibitory concentration

Table S1. Antibiotic susceptibility profile of *Staphylococcus aureus* Newman. WT: wild type.

	MIC [µg/mL]	
	**WT**	**mCherry**
**kanamycin**	4	4
**gentamicin**	0.5	0.5
**tobramycin**	0.5	0.5
**tetracycline**	0.25	0.25
**linezolid**	2	2
**chloramphenicol**	8	64
**erythromycin**	0.25	0.25
**clarithromycin**	0.25	0.25
**ampicillin**	0.125	0.5
**vancomycin**	2	2
**teicoplanin**	1	1
**daptomycin**	2	2
**ciprofloxacin**	0.25	0.25
**sorangicin A**	0.0625	0.0625

1.2 Growth and fluorescence analysis

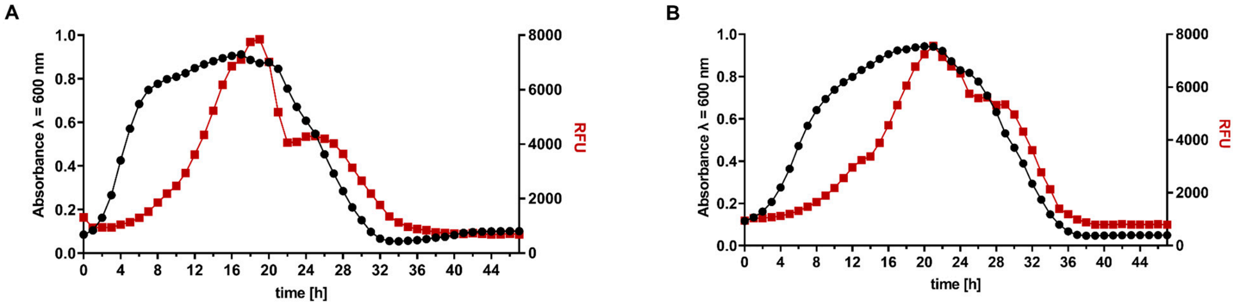

Figure S1. *In vitro* growth and fluorescence analysis of mCherry-expressing *Staphylococcus aureus* Newman at 37 °C (**A**) and at 28 °C (**B**). mCherry expression is delayed by approximately 8–9 hours as compared to *in vitro* growth. Fluorescence intensity (measured as relative fluorescence units, RFU) decreases as bacteria die. Growth as well as RFU were measured using a plate reader (Infinite M200 Pro, Tecan Group Ltd., growth: absorbance at $\lambda = 600$ nm; RFU: $\lambda_{excitation} = 580$ nm, $\lambda_{emission} = 610$ nm).

2. Infection and treatment of zebrafish embryos

Pulled glass capillaries for microinjection were prepared using a micropipette puller (P-1000, Sutter Instrument) with the following settings (**Table S2**).

Table S2. Settings for preparation of microinjection needles.

injection site	properties	heat	pull	velocity	delay	pressure
yolk sac	short, thick	402	80	60	105	235
caudal vein	long, thin	402	80	60	90	100

From 24 hours post fertilization (hpf) on, embryos were maintained in 0.3x Danieau's supplemented with 0.003% N-Phenylthiourea (PTU; FWPTU) to suppress pigmentation. In order to dechorionate embryos, embryos (28 hpf) were incubated for 5 min in pronase (Roche) solution (1 mg/mL in FWPTU) and washed at least three times with fresh 0.3x Danieau's. During the whole course of the injection, embryos were anaesthetized through immersion in 945 μM tricaine (Sigma-Aldrich). For microinjection, a pulled glass capillary connected to an Eppendorf FemtoJet 4x microinjector was filled with the bacterial suspension. The bacterial inoculum contained 2% PVP 40 (Sigma-Aldrich) to improve homogeneity of the suspension. Phenol red solution (Sigma-Aldrich) was added as a tracer dye. The pulled glass capillary was subsequently fixed to the micromanipulator (M-152, Narishige) and the tip was manually cut with a tweezer. The injection volume was then calibrated to 4 nL by injecting single droplets into mineral oil on a microscale slide (Bresser). The anaesthetized embryos (30 hpf) were placed on a 2% agarose mold and injected individually with 4 nL of the bacterial suspension into the yolk sac. Following injection, embryos were placed in 6-well plates (Costar) with fresh FWPTU and maintained at 28 °C. In order to confirm bacterial numbers, an equal volume of bacterial cells was ejected onto TSA supplemented with 10 μg/mL chloramphenicol before and after the injection process. CFU counts were determined after 24 hours of incubation at 37 °C.

Video is provided as separate .avi file.

Video S1. Fluorescence development in zebrafish embryos infected with *Staphylococcus aureus* Newman (≈ 50 CFU). Time-lapse data set was generated using the Celldiscoverer 7 with LSM 900 (Zeiss) together with the 5x/0.35 Plan-APOCHROMAT objective. Images were taken at 1 hour intervals and are shown as inverted images.

Treatment of embryos infected with 50 CFU of mCherry-expressing *S. aureus* Newman was carried out 2 hours post infection (2 hpi) using three different administration routes, namely bath water immersion and microinjection into the caudal vein (CV) as well as the yolk sac. Caudal vein microinjection was performed analogous to yolk injection with the difference that embryos were positioned on a glass slide. Phenol red was used as tracer dye for neutral and negatively charged compounds. Methylene blue (Sigma-Aldrich) was used as tracer dye for positively charged compounds. 10–15 embryos were used per condition. Infected, non-treated embryos served as positive control, whereas non-infected PBS-injected embryos served as negative control. For bath water immersion, infected embryos were maintained in FWPTU containing a defined dose (50–100x MIC) of the antibiotic throughout the whole experiment. The treatment groups and the details of administration are summarized in **Table S3**. Following treatment, embryos were transferred to black 96-well plates (Falcon) with one embryo per well and incubated at 28 °C until 120 hpf. Each treatment experiment was repeated three times.

Table S3. Treatment overview. CS: ClearSol™ Vehicle (LATITUDE Pharmaceuticals Inc.); DMSO: dimethyl sulfoxide; FWPTU: fish water (0.3x Danieau's) + 0.003% N-phenylthiourea (PTU); MB: methylene blue; PBS: phosphate buffered saline; PR: phenol red.

	bath water immersion			microinjection			
	dose [µg/mL]	MIC multiple	solvent	dose [mg/kg]	dose [ng/embryo]	solvent	tracer dye
**ciprofloxacin**	25	100	FWPTU	30	45	PBS	MB
**tetracycline**	25	100	FWPTU	20	30	PBS	MB
**cefazolin**	50	100	FWPTU	30	45	PBS	PR
**linezolid**	100	50	FWPTU/ 1% DMSO	25	37.5	DMSO	PR
**vancomycin**	100	50	FWPTU	20	30	PBS	MB
**sorangicin A**	6.25	100	FWPTU/ 1% DMSO	30	45	CS-2V	PR

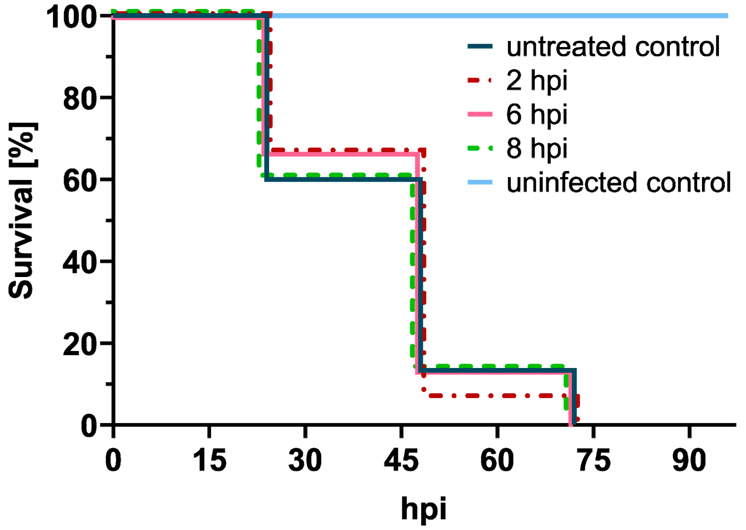

Figure S2. Survival curves of *Staphylococcus aureus*-infected embryos (≈ 50 CFU) treated systemically (caudal vein injection) with 30 ng of vancomycin at different time points (2 hpi, 6 hpi, 8 hpi). Treatment performed at later time points did not impact the efficacy of vancomycin. Non-infected PBS-injected embryos served as negative control.

3. Toxicity of antibiotics

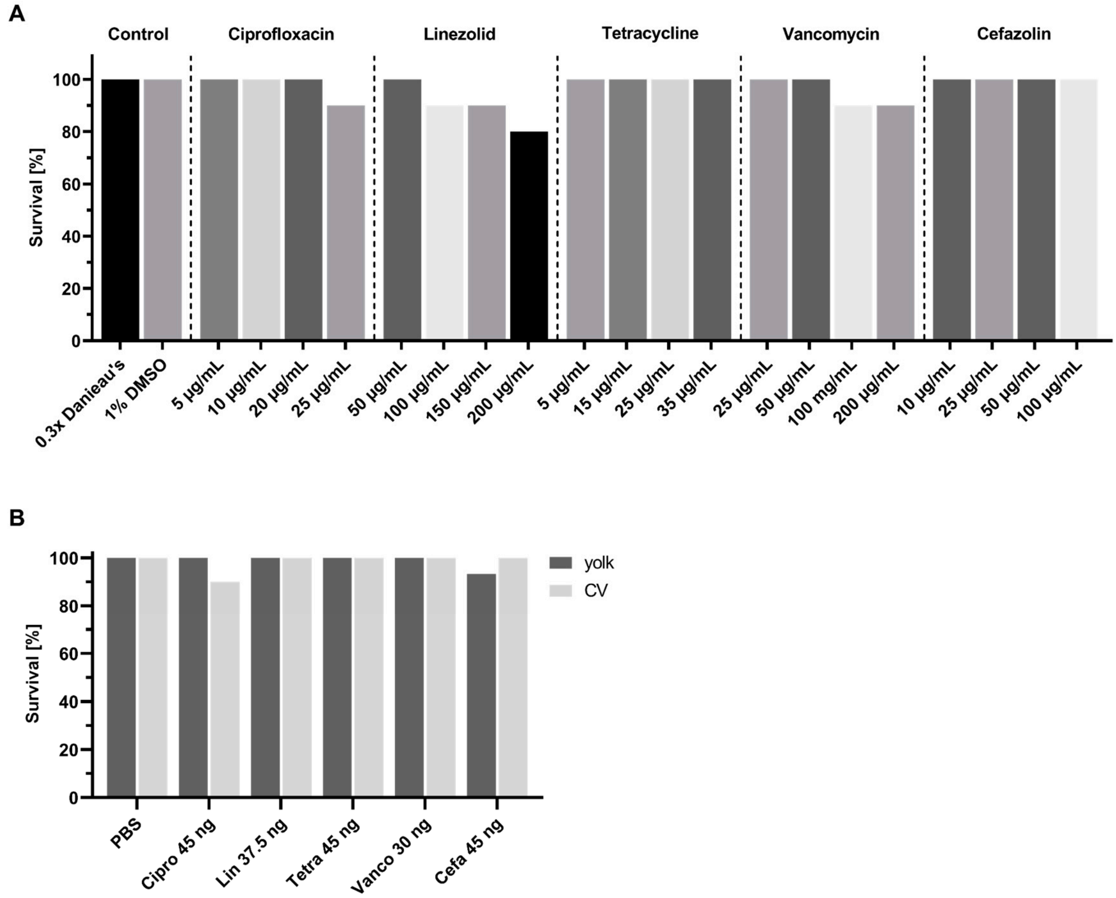

Figure S3. Assessment of toxicity of antibiotics used for validation of the zebrafish embryo model. Survival rates (at 120 hpf) of zebrafish embryos incubated in different concentrations of antibiotics (**A**) or microinjected into the yolk/caudal vein (CV) (**B**) are shown (average of two independent experiments). Cefa: cefazolin; Cipro: ciprofloxacin; Lin: linezolid; Tetra: tetracycline; Vanco: vancomycin.

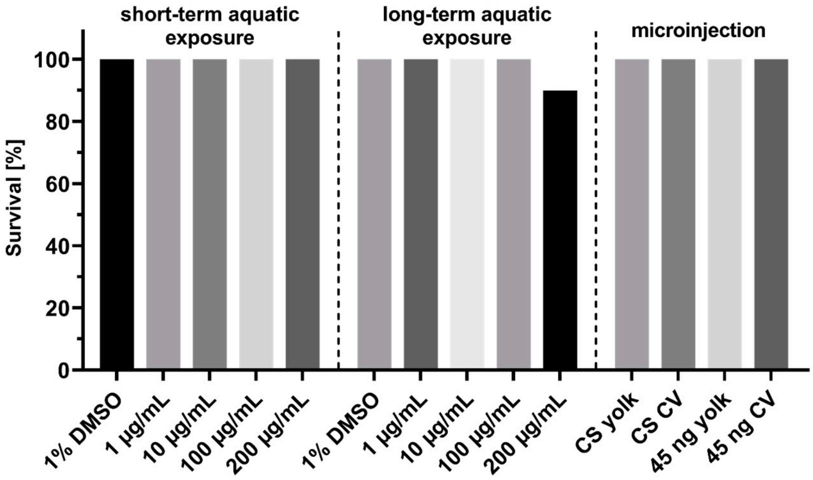

Figure S4. Toxicity assessment of sorangicin A in zebrafish embryos. Survival rates (at 120 hpf) are shown. Short-term aquatic exposure: embryos were exposed from 4 days post fertilization (dpf) to 5 dpf; long-term aquatic exposure: embryos were exposed from 0 dpf to 5 dpf. CV: caudal vein; CS: ClearSol™ Vehicle.

4. Pharmacokinetic evaluation of sorangicin A in mice

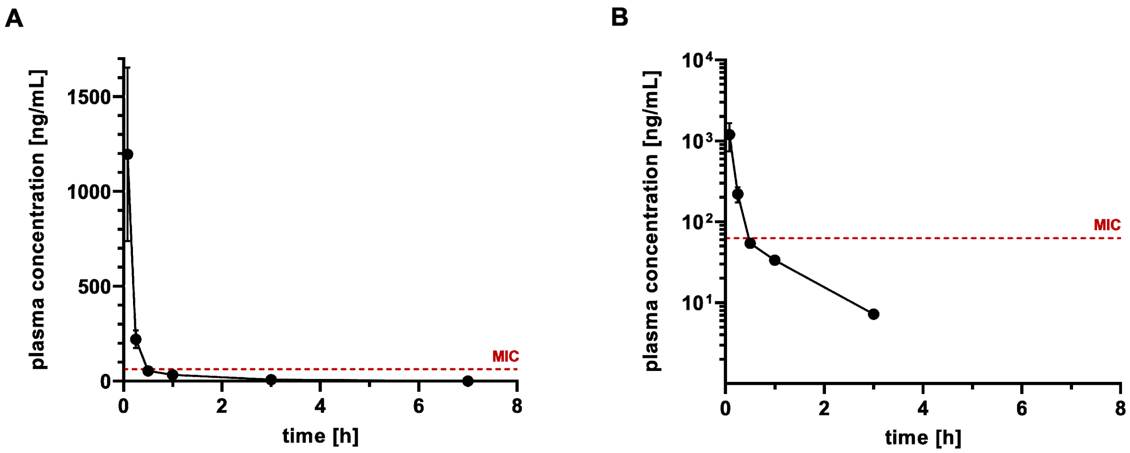

Figure S5. Plasma concentration time profiles of sorangicin A in male CD-1 mice after intravenous administration of 5 mg/kg (mean ± SD, n = 3). **A**: linear scale, **B**: semi-logarithmic scale. The dashed line depicts the minimum inhibitory concentration (MIC) of sorangicin A against *Staphylococcus aureus* Newman.
